# Research Participation Among American Indian and Alaskan Native Individuals Living With Parkinson’s Disease

**DOI:** 10.1155/padi/3207928

**Published:** 2025-12-28

**Authors:** Jacob D. Jones, Yenny Valenzuela, Melissa Pacheco, Lonnie Nelson

**Affiliations:** ^1^ Department of Psychology, California State University, San Bernardino, California, USA, calstate.edu; ^2^ Center on Aging, California State University, San Bernardino, California, USA, calstate.edu; ^3^ Department of Psychology, University of New Mexico, Albuquerque, New Mexico, USA, unm.edu; ^4^ Cancer Prevention Program, Population Health Science Division, Fred Hutchinson Cancer Center, Seattle, Washington, USA

**Keywords:** AIAN, barriers, Parkinson’s disease, research participation, underrepresentation

## Abstract

**Background:**

There is a notable gap in racial and ethnic representation in Parkinson’s disease (PD) research, particularly among American Indian and Alaska Native (AIAN) populations, despite a higher prevalence of PD in these groups. This study investigated research participation among AIAN individuals in terms of perceived access to research opportunities, willingness to participate, and potential concerns about participation.

**Methods:**

Data were obtained from the online Fox Insight (FI) study. A total sample of 4412 individuals who self‐reported their race as White (*n* = 4363) or AIAN (*n* = 49) were selected. The Attitudes and Beliefs Regarding Research and Genetic Testing for PD survey was administered to assess participants’ attitudes and knowledge about the research process, opportunities, and preferences.

**Results:**

A significantly smaller proportion of AIAN individuals (34.7%) reported concurrent or past participation in PD research compared with White non‐AIAN participants (52.9%). Despite this lower participation rate and limited knowledge of research opportunities, a large majority of AIAN individuals (89.8%) expressed a willingness to participate in research. Additionally, both AIAN and White non‐AIAN participants reported similar rates of concerns about research participation. Among AIAN individuals, the most common barriers were distance from research site, transportation, and time commitments.

**Conclusion:**

These findings highlight that low research participation among AIAN individuals may be more associated with low engagement from the research community rather than unwillingness or relatively greater research concerns. Building stronger partnerships with tribal communities and involving community leaders to build trust may improve research representation among AIAN populations.

## 1. Introduction

Underrepresentation of racial and ethnic minorities in clinical research is a well‐known problem in the United States, and it is particularly egregious in research studies of Parkinson’s disease (PD) [1]. A past review of 33 clinical trials of PD found that racial/ethnic minorities made up only 8% of the pool of research participants [2]. This contrasts with the fact that racial/ethnic minorities make up approximately 25% of the US population over 65 years of age. Disparities also exist in observational cohort studies of PD. For example, White non‐Latinos make up approximately 91% of the large multisite study the Parkinson’s Progression Marker Initiative [3]. Representation is slightly improved in an online cohort (the Fox Insight [FI] study), where approximately 86% of the sample were White non‐Latinos [4].

While there are a few existing studies examining research underrepresentation among certain racial/ethnic groups living with PD, there is virtually no research examining participation among American Indian and Alaskan Native (AIAN) populations living with PD. Existing reviews/meta‐analyses of ethnic/racial minority participants in PD clinical trials report the occurrence of Asian, Black, or Hispanic populations [2, 5, 6]. However, data on AIAN individuals is not reported, presumably because either the information was not collected or because researchers grouped AIAN individuals into an “other” group consisting of multiple racial/ethnic categories. Similarly, PD research specifically focused on the AIAN population is limited to medical record review studies of the Indian Health Service to estimate the prevalence of PD [7, 8]. Findings suggest that the prevalence of PD may be higher among the AIAN population (crude prevalence = 144/100,000 and age‐adjusted prevalence = 356/100,000) [7] relative to the Hispanic population (130/1,000,000), White non‐Hispanic population (116/100,000), and African American population (57/100,000) [9]. Estimating the prevalence of PD among the AIAN population is an important first step toward dispelling the myth that low rates of AIAN individuals in PD studies is an accurate reflection of the burden of PD in the AIAN community. However, greater efforts are needed to improve research participation and ultimately clinical care to AIAN communities.

The current study investigates research participation among AIAN individuals in terms of (1) perceived access to research opportunities, (2) willingness to participate in research, and (3) potential concerns about research participation.

## 2. Methods

### 2.1. Participants

This study used data from the online FI study. FI is a national online study of people with and without PD. For up‐to‐date information on the study, visit https://foxinsight-info.michaeljfox.org/insight/explore/insight.jsp. All participants provided consent prior to study activities.

Data were obtained from the FI repository on 11/15/2024. An initial query resulted in 48,212 participants living with PD. The FI study was launched in 2017; however, the Attitudes and Beliefs Regarding Research and Genetic Testing for PD survey (described below) was launched in January 2022. Therefore, we selected individuals who completed the Attitudes and Beliefs Regarding Research and Genetic Testing for PD survey (*n* = 4449). We further selected individuals who self‐reported their race as White (*n* = 4363) or AIAN (*n* = 49), resulting in a total sample size of 4412.

### 2.2. Measures

The Attitudes and Beliefs Regarding Research and Genetic Testing for PD survey was administered to participants at a single time (i.e., cross‐sectional). The survey was created by FI investigators to assess participants’ knowledge and attitudes about the research process, research opportunities, and preferences. We selected items from three domains relevant to participation in PD research, including (1) access‐to/knowledge‐of research opportunities, (2) willingness to participate in various research opportunities, and (3) concerns about various research activities.

### 2.3. Analyses

Pearson chi‐square tests were conducted to examine group differences between AIAN individuals living with PD and White non‐AIAN individuals living with PD for categorical variables. For continuous variables, independent‐samples *t*‐tests were used when data were normally distributed, and Mann–Whitney *U* tests were used when distributions were nonnormal. Group differences were inspected in three single item questions assessing (1) current/past participation in another PD research study (i.e., a PD study besides the FI study), (2) a willingness to participate in PD research even if it did not lead to direct personal benefit, and (3) having any concern about participating in PD research.

Among the AIAN sample only, the relative frequencies are reported for additional items relevant to the domains of access‐to/knowledge‐of research opportunities, willingness to participate in specific research activities, and concerns about specific research activities. The rationale to provide this information among the AIAN sample only is to (1) inform on specific research activities that may be found more or less acceptable among the AIAN sample and (2) identify potential concerns/barriers that researchers can proactively address prior to, during, and after research participation.

## 3. Results

Demographic and clinical characteristics of the participants are available in Table 1. On average, AIAN participants were 48.9% male, age 65.2 years old and with 7.8 years of motor symptoms.

**Table 1:** Demographic and clinical characteristics.

	AIAN (*n* = 49)	White (*n* = 4449)	*t*/Mann–Whitney *Z*	*p* value
Mean age (SD)	65.2 (9.9)	68.5 (8.6)	2.74	0.006
Percent male	48.9%	51.2%	0.31^∗^	0.754
Education	—	—	3.33	< 0.001
Percent high school degree or less	26.7%	6.8%	—	—
Percent some college, no Bachelor’s degree	48.9%	23.2%	—	—
Percent Bachelor’s degree or higher	24.4%	70.0%	—	—
Income	—	—	2.01	0.045
Percent < $35,000	25.6%	13.1%	—	—
Percent $35,000–$75,000	33.3%	29.7%	—	—
Percent > $75,000	41.0%	57.2%	—	—
Mean disease duration: years (SD)	7.8 (5.8)	7.4 (5.4)	0.50	0.619
Mean subjective motor severity (SD)^∗∗^	12.3 (7.6)	9.5 (6.6)	2.71	0.003

Abbreviations: AIAN = American Indian or Alaskan Native, SD = standard deviation.

^∗^Pearson chi‐square.

^∗∗^Assessed via the Unified Parkinson’s Disease Rating Scale, Part II.

### 3.1. Access to Research Opportunities

A total of 34.7% of AIAN individuals reported concurrent or past participation in other research studies in PD. This rate was significantly lower relative to White non‐AIAN participants (Pearson chi‐square = 6.35, *p* = 0.012), of whom 52.9% reported either concurrent or past participation in PD research studies. AIAN and White non‐AIAN participants did not significantly differ in percentage of individuals participating in non‐PD medical research studies (18.4% vs. 11.2%; Pearson chi‐square = 2.52, *p* = 0.112). Regarding knowledge of research opportunities, 32.7% of AIAN participants reported hearing about research opportunities and 40.8% knew where/how to find a research study.

### 3.2. Willingness to Participate in Research

Despite relatively low participation in PD research and knowledge of research opportunities, an overwhelming majority of AIAN individuals reported a willingness to participate in research (89.8%). This response rate was not significantly different relative to White non‐AIAN individuals (Pearson chi‐square = 1.83, *p* = 0.400), of whom 82.6% reported a willingness to participate in research.

AIAN individuals’ willingness to participate in specific research tasks is shown in Table 2. More than half of the sample was willing to participate in activities common to noninvasive observational studies, such as online surveys, clinic interviews/surveys, or brain imaging. Regarding potential treatment studies, exercise and oral/skin patch/inhaler medication were acceptable to approximately half of the participants. Brain surgery and taking medication via intestinal tube were the least acceptable activities (only 10.2% reported these activities as acceptable).

**Table 2:** Willingness to participate in research activities among AIAN participants.

Survey content	Percent willing to participate (%)
Online surveys	93.9
Provide a saliva sample	83.7
Complete interview via computer/smartphone	73.5
Provide a blood sample	71.4
Complete interview in a clinic	63.3
Complete surveys in a clinic	59.2
Exercise, walk, or run	59.2
Have a brain scan/MRI	57.1
Take oral medication	55.1
Telephone surveys	53.1
Take medication via skin patch	49.0
Take medication via inhaler	46.9
Take intravenous medication	34.7
Outpatient procedure (e.g., lumbar puncture or skin biopsy)	34.7
Take medication via injection	32.7
Take medication via intestinal tube	10.2
Have brain surgery	10.2
Not willing to do any of the above	4.1

Abbreviations: AIAN = American Indian or Alaskan Native, MRI = magnetic resonance imaging.

### 3.3. Concerns About Research Participation

The majority of both AIAN (85.7%) and White non‐AIAN (78.0%) participants reported at least one concern about participating in research; a difference which was not statistically significant (Pearson chi‐square = 1.70, *p* = 0.192).

The distance from residence to the research site was the most commonly reported concern among AIAN individuals (67.3%; Table 3), followed by transportation (42.9%) and the time commitment (24.5%).

**Table 3:** Concerns among AIAN and White participants.

Survey content	Percent of AIAN participants endorsing the concern (%)	Percent of white participants endorsing the concern (%)
Distance from residence to the research center	67.3	57.2
Transportation to/from research center	42.9	31.3
Time commitment	24.5	26.4
Complex instructions or consent forms	22.4	12.3
Effort involved in participation	20.4	24.9
Privacy of my health information	20.4	12.6
Any concerns about genetic testing	16.3	13.5
Lack of knowledge about genetic testing	10.2	4.7
Concerned about privacy of genetic testing results	10.2	5.9
Researchers may not understand/respect my religious/cultural beliefs	6.1	2.1
Research participants may be different than me	2.0	1.8
Researchers may not speak my preferred language	2.0	3.8

Abbreviation: AIAN = American Indian or Alaskan Native.

## 4. Discussion

The findings indicate a clear discrepancy between a high (approximately 90%) willingness to participate in research studies but dramatically lower (< 41%) engagement or knowledge of research opportunities among AIAN individuals living with PD. AIAN individuals were particularly willing to participate in noninvasive observational study activities like online surveys, clinic interviews, and brain imaging. However, many cited barriers such as distance to research sites and transportation issues.

Lower research engagement of AIAN individuals living with PD reflects broader systemic issues in PD healthcare, where racial and ethnic minorities often face limited access to care and underrepresentation in clinical studies. A review of 239 clinical trials of PD conducted over 22 years revealed that only 17% included race and ethnicity data, and of the 7481 participants enrolled, just 8% were non‐White [2]. Additionally, after a PD diagnosis, only 58% of people are seen by a neurologist, with non‐White patients being less likely to receive neurologic care [1, 10]; unfortunately, data on AIAN participants were not separately reported in this study. Since research recruitment commonly occurs in clinical settings, disparities in clinical care will continue to contribute to underrepresentation in research. Similarly, a lack of representation of AIAN individuals in PD research compounds the risk of adverse clinical outcomes (e.g., hip fractures and mortality) [1], as research findings may not account for the unique environmental, socioeconomic, and cultural factors that affect this population and other minority populations [6]. For example, PD may manifest differently in AIAN individuals, and tailored interventions may be less effective or acceptable if they are developed primarily from research conducted on predominantly White populations [11].

Access to specialty care is another critical factor that may influence both clinical outcomes and opportunities for research participation. Even within the general PD population, barriers to accessing healthcare services are well documented. Zaman et al. [12] conducted a scoping review which found that people with PD face both person‐level (e.g., low health literacy and communication challenges) and system‐level (e.g., limited service availability and strained healthcare infrastructure) barriers that reduce access to appropriate care. These issues are likely magnified in AIAN communities, where older adults face additional challenges in securing specialty care. Sommerfeld et al. [13] found that older American Indian adults perceived factors like limited elder‐specific transportation services, the need to travel long distances for care, and the lack of community health programs offering transportation, as well as challenges with scheduling, provider relationships, and insecurity from lack of knowledge about navigating the healthcare system as significant barriers to healthcare access. Because specialty clinics often serve as primary hubs for research recruitment, inequitable access to such clinics may further contribute to the persistent underrepresentation of AIAN individuals in PD research.

In line with these broader specialty care challenges, distance and transportation were prominent concerns among AIAN participants in our study. A high percentage of AIAN individuals expressed concerns over distance from residence to research center, as well as concerns over transportation to/from research center. Many Native communities in remote areas face challenges accessing healthcare due to limited transportation options. While a majority of AIAN individuals currently reside in urban areas, significant health disparities persist, driven by factors such as high rates of poverty, communication barriers, and long distances from healthcare services [14]. Older AIAN adults are the most affected by these barriers as most tend to rely on family/friend caregivers to provide transportation. Therefore, implementing transportation services, or at least budgeting for transport to and from study visits as part of the research study, is essential to promoting research participation within this group.

Among AIAN individuals living with PD, a demanding/intense time commitment was the third most common concern. On one hand, the fact that approximately three‐quarters of AIAN participants did not endorse this concern speaks to a strong willingness to participate in research among AIAN individuals living with PD. On the other hand, many research studies require participants to undergo specific tests, surveys, or interviews that may be lengthy and may require multiple visits, which might be a deterrent of research participation, especially among older AIAN adults. Patients are likely to weigh the potential benefits of observational studies and clinical trials against factors such as long study durations, demanding medical appointments, and high transportation costs, which can burden participants and discourage research involvement [15]. Offering reasonable incentives and flexibility with participants’ schedules could help encourage both participation and retention among older AIAN adults.

Building partnerships with tribal communities, involving community leaders in the research process, and demonstrating cultural sensitivity are some approaches to encourage research participation among older AIAN adults (Table 4). In order to better meet the needs of indigenous peoples with chronic disease, Wali et al. [18] suggested adopting community engagement strategies, such as involving community members at various stages of the research process (e.g. before, during, and after intervention/data collection implementations) and gaining community approval of the study. Furthermore, moving research sites to local communities, addressing transportation issues, and enhancing staff sensitivity to minority needs are other suggestions to improve AIAN research recruitment and participation [2]. Knight et al. [17] and Brockman et al. [16] have highlighted that community‐based participatory research and community outreach approaches (e.g., events to promote recruitment) are effective methods for reducing barriers to participation.

**Table 4:** Actionable strategies to improve research engagement among AIAN populations.

Actionable strategy	Description/rationale	Supporting references
Build partnerships with tribal communities	Establish long‐term, trust‐based collaborations with tribes and community organizations before study initiation; obtain tribal or community approval for participation	[16, 17]
Engage community and tribal leaders	Involve leaders throughout planning, recruitment, and dissemination to ensure relevance, transparency, and shared ownership	[16, 18]
Demonstrate cultural sensitivity and adapt interventions to local context	Adapt study materials, consent processes, and communication styles to reflect AIAN cultural values, language, and community norms	[16, 18]
Localize research activities	Conduct study visits and data collection within or near AIAN communities to improve accessibility and minimize travel burden	[17]
Address transportation and logistical barriers	Provide transportation assistance or budget for travel and lodging to enable participation	[17]
Train research staff in cultural competence	Train staff with AIAN history, sovereignty, communication norms, and local customs to build trust and reduce misunderstanding	[16, 18]
Use community‐based participatory research (CBPR) and coleadership approaches	Engage AIAN community members as partners or coleaders across all stages of research, from design to dissemination	[16, 17]
Enhance community outreach and feedback	Maintain open communication and feedback loops through events, workshops, and advisory boards to sustain engagement and transparency	[16, 17]

Abbreviation: AIAN = American Indian or Alaskan Native.

To the best of our knowledge, this is the first study to investigate research participation among AIAN individuals living with PD. While this study shines a light on possible steps for advancing research representation, there are limitations. There is likely to be selection bias in this sample. By definition, all participants were willing to at least enroll in the FI online study of PD. Therefore, the current participant’s attitudes about research participation may not reflect the entire population of AIAN individuals living with PD. The sample size of AIAN participants is relatively limited (*n* = 49). However, data about AIAN participants typically is not reported, or AIAN participants make up an extremely small percent of participants in PD studies. We hope these preliminary findings increase research opportunities and engagement among AIAN communities and ultimately lead to more refined investigations in research participation and clinical outcomes.

Overall, AIAN individuals living with PD have been poorly represented in clinical research. This underrepresentation may reflect limited scientific engagement in AIAN communities rather than low motivation or an unwillingness to participate in research among AIAN individuals living with PD.

## Conflicts of Interest

The authors declare no conflicts of interest.

## Funding

The Fox Insight Study (FI) was funded by the Michael J. Fox Foundation for Parkinson’s Research.

## Data Availability

The data that support the findings of this study are openly available in FoxDen at https://foxden.michaeljfox.org/insight/explore/login.jsp.
